# Hypothermic neuroprotection during reperfusion following exposure to aglycemia in central white matter is mediated by acidification

**DOI:** 10.14814/phy2.14007

**Published:** 2019-03-04

**Authors:** Angus M. Brown, Richard D. Evans, Paul A. Smith, Laura R. Rich, Bruce R. Ransom

**Affiliations:** ^1^ School of Life Sciences Queens Medical Centre University of Nottingham Nottingham United Kingdom; ^2^ Department of Neurology School of Medicine University of Washington Seattle Washington

**Keywords:** Aglycemia, glucose, hypothermia, neuroprotection

## Abstract

Hypoglycemia is a common iatrogenic consequence of type 1 diabetes therapy that can lead to central nervous system injury and even death if untreated. In the absence of clinically effective neuroprotective drugs we sought to quantify the putative neuroprotective effects of imposing hypothermia during the reperfusion phase following aglycemic exposure to central white matter. Mouse optic nerves (MONs), central white matter tracts, were superfused with oxygenated artificial cerebrospinal fluid (aCSF) containing 10 mmol/L glucose at 37°C. The supramaximal compound action potential (CAP) was evoked and axon conduction was assessed as the CAP area. Extracellular lactate was measured using an enzyme biosensor. Exposure to aglycemia, simulated by omitting glucose from the aCSF, resulted in axon injury, quantified by electrophysiological recordings, electron microscopic analysis confirming axon damage, the extent of which was determined by the duration of aglycemia exposure. Hypothermia attenuated injury. Exposing MONs to hypothermia during reperfusion resulted in improved CAP recovery compared with control recovery measured at 37°C, an effect attenuated in alkaline aCSF. Hypothermia decreases pH implying that the hypothermic neuroprotection derives from interstitial acidification. These results have important clinical implications demonstrating that hypothermic intervention during reperfusion can improve recovery in central white matter following aglycemia.

## Introduction

It is widely accepted that hypothermia affords protection during ischemia, that is, combined anoxia and hypoglycemia, in the central nervous system with the optimal degree of protection occurring with hypothermia of 5 to 8°C (Wassink et al. 2018); hyperthermia augmenting injury (Greer et al. 2008; Campos et al. 2013). Therapeutic hypothermia has also successfully been used to spare neurological function following cardiac arrest (Hypothermia after Cardiac Arrest Study, 2002; Bonaventura et al. 2016) and as a result of severe hypoxic‐ischemic encephalopathy (Azzopardi et al. 2012), but had no beneficial effect on patients subjected to severe traumatic brain injury (Cooper et al. 2018), suggesting hypothermia's benefits are only manifest in injuries resulting from metabolic disruption. From the clinical perspective this is valuable information since, given limited availability of neuroprotective therapies, lowering patients’ temperature can be a therapeutically viable means of limiting brain damage following stroke (Polderman 2004a,b).

The majority of available information regarding hypothermic neuroprotection in the CNS concerns ischemic injury to gray matter regions, notably the hippocampus and the cortex (Greiner et al. 1998; Colbourne et al. 1999). However, in the adult human white matter constitutes 50% of the brain volume (Zhang and Sejnowski 2000), and all ischemic events involve not only gray matter but the underlying white matter (Dirnagl et al. 1999). Indeed lacunar infarcts are exclusively white matter injuries (Fisher 1982). There is a paucity of information regarding any putative neuroprotective effects of hypothermia on aglycemic injury. Patients suffering from type 1 diabetes are at risk of systemic hypoglycemia as a result of mismatching insulin administration against prevailing blood glucose levels (Frier et al. 2014). The brain is exquisitely sensitive to shortfalls in delivery of glucose and responds with a range of autonomic signals (Cryer 2015). The damage incurred by aglycemia in the brain is region specific with the dentate gyrus most sensitive based on electron microscopic morphological analysis, and the cerebellum and brainstem spared (Auer 2004). However, white matter is also sensitive to hypoglycemia (Ma et al. 2009; Johkura et al. 2012). There appear to be two phases of injury associated with hypoglycemia. The first phase occurs following the loss of electrical excitability, which coincides with a flat EEG (Auer et al. 1984). The ATP production decreases leading to membrane depolarization and aspartate‐mediated activation of NMDA receptors (Yang et al. 2014), opening of voltage gated Ca^2+^ channels and reversal of the Na^+^‐Ca^2+^ exchanger (Brown et al. 2001), all of which lead to toxic Ca^2+^influx into axons. The second phase is associated with reperfusion of glucose, as occurs during medical intervention, which offers an opportunity for therapeutic intervention. In rat gray matter, the majority of the hypoglycemia induced injury is attributable to activation of NADPH oxidase during reperfusion (Suh et al. 2007), suggesting a potentially viable therapeutic strategy is to impose hypothermia during reperfusion, whereas the mitochondrial permeability transition pore contributes to reperfusion injury in cardiac myocytes (Morciano et al. 2017). Prior data from rodent central white matter revealed there is no advantage in imposing hypothermia following exposure to anoxia (Stys et al. 1992a), but no equivalent information is available for aglycemic injury.

In this study we used the established model of CNS white matter injury, the mouse optic nerve (MON). We demonstrated a linear relationship between the degree of injury incurred by the MON and duration of exposure to aglycemia, which served as a baseline against which to compare test data. Imposing hypothermia during the entire experiment (aglycemia exposure and reperfusion) improved recovery compared with control experiments carried out at 37°C. Restricting exposure of the MON to hypothermia exclusively to the reperfusion phase resulted in equivalent CAP recovery to that during combined aglycemia and reperfusion, similar to the result reported in gray matter (Suh et al. 2007). The effect appeared to be mediated via hypothermic acidification of the aCSF suggesting that plausible strategies to protect the brain from hypoglycemia‐induced injury are to impose hypothermia during reperfusion of glucose or alter the patient's blood pH via intubated gases.

## Materials and methods

### Ethical approval

All experiments were approved by the University of Nottingham Animal Care and Ethics Committee, were carried out in accordance with the Animals (Scientific Procedures) Act 1986 under appropriate authority of establishment, project and personal licenses, and conform to the principles and regulations described in the Editorial by Grundy (2015). Experiments were performed on male CD‐1 mice (weight 28–35 g, corresponding to 30–45 days of age) purchased from Charles River Laboratories (Margate, Kent, CT9 4LT, UK). Mice were group housed with *ad libitum* access to food and water, and maintained at 22–23°C on a 12:12 h light‐dark cycle. Mice were killed by Schedule 1 cervical dislocation; death was confirmed by permanent cessation of the circulation. A total of 74 mice were used. In total 61 MON recordings were made. The authors understand the ethical principles under which The Journal of Physiology operates and declare this work complies with this animal ethics checklist.

Adult male CD‐1 mice were killed by Schedule 1 methods (see above). Optic nerves were dissected free, cut at the optic chiasm and behind the orbit, gently freed from their dural sheaths, placed in an interface perfusion chamber (Model BSC‐BU, Medical Systems Corp, Greenvale, NY, USA), and maintained at 37°C with superfusion of control artificial cerebrospinal fluid (aCSF) containing (in mM): NaCl 126, KCl 3.0, CaCl_2_ 2.0, MgCl_2_ 2.0, NaH_2_PO_4_ 1.2, NaHCO_3_ 26 and glucose 10. Bath pH was changed from 7.45 to 7.55 by increasing NaHCO_3_ to 35 mmol/L and decreasing NaCl to 117 mM. A temperature change from 37°C to 27°C increases CO_2_ solubility and is calculated to acidify aCSF from 7.40 to 7.29 (see below), confirmed by experimental measurement. The perfusion chamber was continuously aerated with a humidified gas mixture of 95% O_2_/5% CO_2_. A feedback heating unit (Model TC 202A, Harvard Apparatus, UK) maintained the temperature at the desired levels. The slow time course of the temperature changes illustrated in Figure 4A highlights the inertia in heating the chamber, thus to accelerate the transition of temperature changes to within 5 min (see Fig. 6A) we drained the reservoir of water via a sump in the base of the unit and rapidly and repeatedly injected water at the desired temperature into the reservoir until the stable, desired temperature was reached.

### Electrophysiology

Suction electrodes back‐filled with the appropriate aCSF were used for stimulation and recording. One electrode was attached to the rostral end of the nerve for stimulation and the second suction electrode was attached to the caudal end of the nerve to record the compound action potential (CAP), thus all recordings were orthodromic. A WPI Isostim A320 constant current stimulator (WPI, Astonbury Farm Business Centre, Stevenage, UK) was used to generate a stimulus of 30 μs duration and its strength adjusted to evoke the maximum CAP possible and then increased another 25% (i.e. supramaximal stimulation, Stys et al. 1991). During an experiment, the supramaximal CAP was elicited every 30 sec. The signal was amplified 1000x by a Stanford Research Systems Preamplifier (SR560, Stanford Research Systems, Inc., 1290‐D Reamwood Ave., Sunnyvale, CA 94089, USA), low pass filtered at 10 kHz and acquired at 20 kHz (Digidata 1320A with Clampex 9.2, Molecular Devices, Wokingham, UK). Optic nerve axon function was monitored quantitatively as the area under the supramaximal CAP. The area under the CAP represents the best measure of the number of active axons because currents generated by individual axons within a fiber tract are considered to sum linearly (Cummins et al. 1979; Stys et al. 1991). The curve fitting routine for detecting latency to CAP failure has previously been described (Wender et al. 2000; Brown 2006).

### Oxygen consumption

The O_2_ consumption was measured polarographically using Clark oxygen electrodes (Rank Brothers, Bottisham, UK) in a 1 mL sealed volume of aCSF, constantly stirred and maintained at 37°C with a Haake Water circulator. For each experiment, 2 MONs were placed in each chamber and allowed to equilibrate for 30 min. For O_2_ measurements the chamber was then sealed from the atmosphere with a plunger (closed system). Care was taken to expel all gas bubbles and the stirring speed checked to ensure that the MONs were suspended. The partial pressure of O_2_ (pO_2_) was measured at a polarographic voltage of −0.6 V with electrodes calibrated at 100% saturation in aCSF bubbled with 95% O_2_/5% CO_2_, and at 0% by addition of Na_2_S_2_O_4_. aCSF was bubbled with 95% O_2_/5% CO_2_ for 2 h at the test temperature to ensure solution saturation with gas. The rate of O_2_ consumption was measured as the change in pO_2_ over a period of time, which being linear, was the pO_2_ slope. The pO_2_ was converted to molarities based on assumptions of solubility of O_2_ at 35°C being 19.82 μmoles per mole H_2_O, and 22.90 μmoles per mole H_2_O at 25°C (Lide 2006), and corrected for tissue weight.

### Temperature dependence of CO_2_ solubility

The buffer system catalyzed by carbonic anhydrase:

$${\text{CO}}_{2\text{aq}}+H_{2O}\Leftrightarrow H_{2}{\text{CO}}_{3}\Leftrightarrow H^{+}+{{H\;}_{3}}^{-}$$

can be simplified since the concentration of H_2_CO_3_, carbonic acid, is very low compared with the other components:

$${\text{CO}}_{2}+H_{2}O\Leftrightarrow H^{+}+{{\text{HCO}}_{3}}^{-}$$

where pH = −log_10_ [H^+^]

From the Henderson‐Hasselbalch relationship

$$\mathrm{pH}=pK_{a}-\log\frac{\left[\mathrm{acid}\right]}{\left[\mathrm{base}\right]}$$

pK_a_ for CO_2_/HCO_3_^−^ = 6.1 at 37°C at physiological ionic strength (Chang 1981).

$$\text{[Acid]}={{\text{[CO}}_{2}]}_{\mathrm{aq}}$$

[CO_2_]_aq =_ p(CO_2_) x K^T^ where p(CO_2_) is the partial pressure of CO_2_ in the atmosphere and K^T^ is the solubility coefficient for CO_2_ at T˚C in mol atm^−1^ dm^−3^

K^298^: solubility coefficient for CO_2_ at 25°C (298 K) = 0.034 mol atm^−1^ dm^−3^ (Haynes 2010).

$$K^{T}=K^{298}\exp[C^{}*\left(\frac{1}{273+T}\right)-\left(\frac{1}{298}\right)]$$

where K^T^ is the solubility coefficient for CO_2_ at T˚C and C = 2400 for CO_2_.

If follows that K^37^ = 0.025 mol atm^−1^ dm^−3^, thus [CO_2_]_aq_ = 0.05 x K^37^ at 5% CO_2_ at 37°C

[CO_2_]_aq _= 0.00125 M

[Base] = [HCO_3_^−^] in aCSF =^ ^0.026 M

pH = 7.40 at 37°C, pH = 7.29 at 27°C and pH = 7.46 at 42°C

### Transmission Electron Microscopy (TEM)

MONs were dissected as described above, laid out on cardboard and then immediately fixed in 2% glutaraldehyde and 2% paraformaldehyde in 0.2 mol/L phosphate buffer overnight and postfixed in 1% osmium tetroxide for 30 min. They were dehydrated in a graded ethanol series and embedded in Transmit low viscosity resin (TAAB). Semi thin sections were cut at 0.5 μm, stained with toluidine blue and photographed using a Leica DM400B light microscope with color digital camera and Openlab darkroom software. Ultrathin sections (70–90 nm) were prepared using a Reichert‐Jung Ultracut E ultramicrotome and mounted on 100 hexagonal copper grids. They were contrasted using Uranyl acetate and Lead Citrate and viewed using a JEOL 1010 TEM operated at 80 kV with digital image acquisition.

### Computer simulation

The simulations were carried out using NEURON 7.4 (Hines and Carnevale 1997). The cell builder module was used to construct a model of a myelinated axon consisting of alternating myelinated internodal regions (INR) and unmyelinated nodal compartments, with 26 of the former, and 25 of the latter as previously described (Kolarik et al. 2013). The morphological dimensions, and passive measures of conductance, resistance, and capacitance were based on data from corpus callosum axons and oligodendrocytes (Bakiri et al. 2011), and were integrated to construct the basic axon model. The voltage dependent conductances (Richardson et al. 2000), upon which action potential conduction depends, were incorporated into the nodal compartments. Simulated action potentials were computed using backward Euler integration with a time step of 0.01 msec. The temperature dependence of the rate constants were based on previously published Q_10_ values (Richardson et al. 2000). Action potentials were evoked by a constant current pulse, and recorded 10 INRs distant from the site of current injection.

### Lactate Biosensors

Lactate biosensors (Frenguelli et al. 2003) were obtained from Sarissa Biomedical Ltd (Coventry, UK). In practice, the lactate signals were so large, relatively, that subtraction of the null signal had no effect on the signal amplitude. The lactate biosensors (25 μm diameter and 500 μm length) were pressed against the optic nerve and allowed to equilibrate for 30 to 60 min prior to experiments. At the end of a recording the biosensors were calibrated using lactate concentrations of 10 μmol/L and 100 μmol/L.

### Statistical analysis

**Figure 1:**
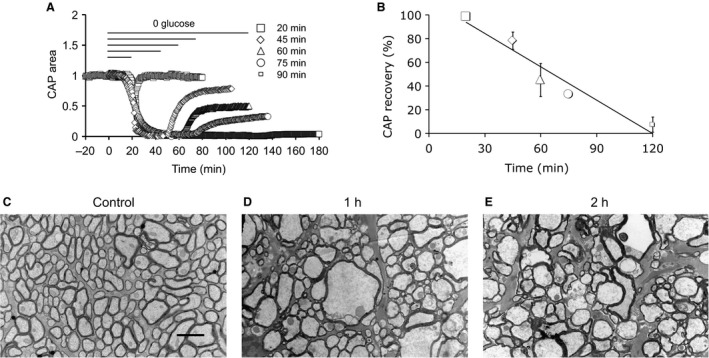
CAP recovery is dependent upon duration of exposure to aglycemia. (A) CAP area versus time for MONs exposed to aglycemia for 20, 45, 60, 75, or 120 min, followed by a 60‐minute recovery period in control aCSF. All experiments carried out at 37°C. The period from −20 to 0 min indicates the baseline prior to introduction of 0 glucose, and similar baseline periods are shown in Figure 2A and C, Figure 3A and B and Figure 6A and B. (B) The CAP recovered to 98.5 ± 2.9% (*n* = 5), 78.2 ± 7.3% (*n* = 5), 45.0 ± 13.9% (*n* = 5) 33.0 ± 2.1% (*n* = 4), or 7.3 ± 6.4% (*n* = 5) after 20 min, 45 min, 60 min, 75 min, or 120 min of aglycemia, respectively, a linear relationship (Pearson correlation: *R* ^2^ = 0.94, *P* = 0.007). (C) Electron micrograph of a control MON cut in transverse section illustrates axons considered normal based on their robust appearance. Scale bar = 2 μm also applies to D and E. (D) MONs exposed to 1 h of aglycemia followed by 1 h reperfusion with control aCSF display swollen axons with appearance of intraaxonal compartmentalization, ruptured myelin, and swollen mitochondria. (E) MONs exposed to 2 h of aglycemia display extreme injury including multiple swollen axons, loss of myelin wraps, appearance of large intraaxonal vacuoles, and loss of axolemma.

**Figure 2:**
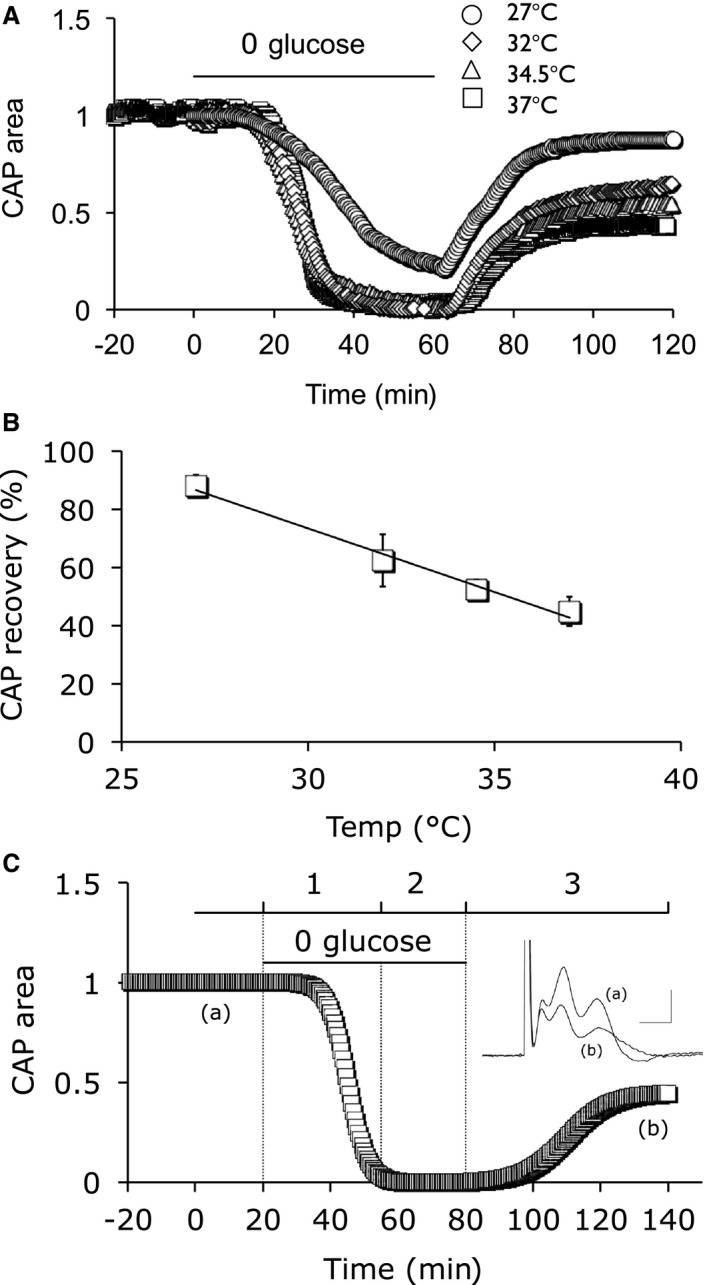
Ambient temperature determines CAP recovery after a 1 h period of aglycemia followed by 1 h reperfusion in control aCSF. (A) MONs were exposed to 1 h of aglycemia followed by a 1 h recovery period in control aCSF at 27°C, 32°C, 34.5°C, or 37°C. (B) The CAP recovered to 44.8 ± 5.0% (*n* = 7), 52.5 ± 3.5% (*n* = 5), 62.3 ± 8.9% (*n* = 8) or 87.9 ± 3.7 (*n* = 6) at 37°C, 34.5°C, 32°C or 27°C, respectively, a linear relationship (Pearson correlation: *R* ^2^ = 0.99, *P* = 0.006). (C) Schematic of the multiple phases of aglycemia: baseline, (1) glycogen usage whose exhaustion initiates CAP failure, (2) CAP failure in the continued presence of aglycemia, and (3) reperfusion phase on reintroduction of control aCSF. Inset illustrates CAPs from the baseline period (a) and after exposure to aglycemia and recovery in 10 mM glucose aCSF (b). Scale bars 0.5 msec and 1 mV.

**Figure 3:**
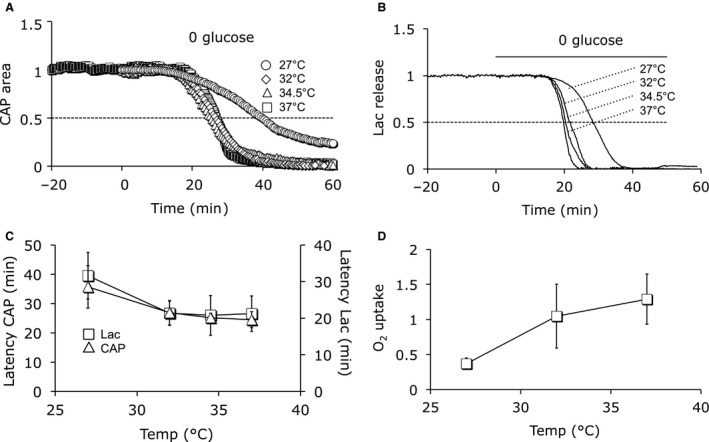
Temperature dependence of aglycemia induced CAP failure and lactate release. (A) Aglycemia induced CAP failure that was temperature dependent. The CAP was maintained for 26.5 ± 6.2 min (*n* = 7), 26.0 ± 6.8 min (*n* = 8), 26.7 ± 4.2 min (*n* = 5), or 39.5 ± 7.9 min (*n* = 6) at 37°C, 34.5°C, 32°C or 27°C, respectively. (B) Lactate release from MONs decreased on exposure to aglycemia in a temperature dependent manner: lactate fell to 50% of baseline values after 19.6 ± 2.2 min (*n* = 3), 20.1 ± 1.2 min (*n* = 4), 21.5 ± 3.3 min (*n* = 3), or 28.6 ± 5.8 min (*n* = 3) at 37°C, 34.5°C, 32°C or 27°C, respectively. (C) Plots of latency to CAP failure and latency to lactate fall did not show any discernable temperature dependence between 37°C and 32°C, but both parameters were increased at 27°C. (D) Oxygen uptake (μmoles O_2_ g^−1^ min^−1^) in MONs was 0.37 ± 0.08 μmoles O_2_ g^−1^ min^−1^ (*n*  = 2), 1.04 ± 0.45 μmoles O_2_ g^−1^ min^−1^ (*n*  = 2), or 1.29 ± 0.35 μmoles O_2_ g^−1^ min^−1^ (*n*  = 2) at 27°C, 32°C or 37°C, respectively (Pearson correlation: *R* ^2^ = 0.93, *P* = 0.04).

Data are presented as the mean and the standard deviation. The graphs showing CAP area versus time are averaged traces of all observations recorded, with the recovery determined as the ratio of the CAP area measured in the last 5 min of the recovery period versus the baseline CAP area. Statistical analysis was carried out using Pearson correlation (Figs. 1B, 2B, 3B) and 2‐way ANOVA (Fig. 6C). Statistical significance was set at *P* < 0.05 and all statistical analyses were performed using GraphPad Prism 7.0 (GraphPad Software, Inc., La Jolla, CA, USA).

## Results

### Duration of exposure to aglycemia determined CAP recovery

We have previously shown that exposure to aglycemia caused delayed failure of the stimulus evoked CAP in MONs with partial recovery of the CAP possible, but dependent upon duration of aglycemia exposure (Baltan Tekkök et al. 2003; Yang et al. 2014). In this study the partial CAP recovery was further studied by exposing MONs to aglycemia for a wider range of durations (from 20 min to 120 min). In MONs exposed to 20 min of aglycemia the CAP started to fall but was fully rescued by reperfusion with control aCSF. However, in MONs exposed to 45 min of aglycemia the CAP fell to zero where it remained for about 10 min before reperfusion partially rescued the CAP, suggesting the injury process commences after the CAP has completely failed, rather than at the onset of CAP failure. Increasing the duration of exposure to aglycemia resulted in correspondingly less CAP recovery (Fig. 1A and B).

Electron micrographic analysis of axons was carried out to determine if the loss of axon conduction correlated with morphological detection of pathological changes as a result of exposure to aglycemia. MONs under control conditions (Fig. 1C) showed good preservation and displayed squamous or elliptical profiles, as well as the relatively ordered morphology previously reported in control rodent optic nerve axons (Garthwaite et al. 1999; Allen et al. 2006). MONs exposed to 1 h aglycemia followed by a 1 h reperfusion period in 10 mM glucose aCSF displayed clear signs of axon swelling, intraaxonal compartmentalization, disrupted myelin and swollen mitochondria (Fig. 1D), effects that were more pronounced after exposure to 2 h of aglycemia followed by reperfusion (Fig. 1E).

### Temperature affected aglycemia induced injury

MONs were bathed in 10 mmol/L glucose for a baseline period of up to 30 min, then exposed to aglycemia for 1 h, and allowed to recover in 10 mmol/L glucose aCSF for 1 h at 27°C, 32°C, 34.5°C or 37°C for the duration of the experiment (Fig. 2A). In MONs maintained at 32°C, 34.5°C or 37°C the CAP fell at roughly the same time, whereas at 27°C the failure was delayed (see Fig. 3A and C). The degree of CAP recovery upon reperfusion of aCSF containing 10 mM glucose was temperature dependent, the MONs incubated at the lowest temperature exhibiting the highest degree of recovery (Fig. 2B), although if one compares the recovery of the CAP from the end of the period of aglycaemia to the end of the reperfusion period, the values at 27°C would be lower due to the CAP not having fallen to zero by the end of the period of aglycaemia.

The effects of aglycemia can be divided into phases as shown in Figure 2C. The initial phase denotes the baseline CAP against which the post‐aglycemia CAP area can be compared to determine the degree of injury incurred by the MON. Introduction of aglycemia signifies the onset of Phase 1. The CAP was initially fully maintained as a result of glycogen metabolism but then fell to zero once the glycogen was exhausted (Baltan Tekkök et al. 2003). Based on the data shown in Figure 1A and B we presume that no injury is incurred by the tissue during this phase. The failure of the CAP signaled the onset of Phase 2. Phase 3 denotes the reperfusion of control aCSF and (partial) recovery of the CAP. In the subsequent experiments we sought to determine the effects of temperature during phase 3.

### Effect of temperature on aglycemia induced CAP failure and lactate release

The effect of temperature on the latency to aglycemia‐induced CAP failure was assessed by measuring the latency to 50% CAP area compared with its baseline value. CAP failure occurred at similar times at 32°C to 37°C but was increased at 27°C (Fig. 3A and C). We have previously shown that lactate measured at the pial‐glial boundary of the MON with enzyme electrodes offers a convenient proxy for the supply of glycogen‐derived lactate within the tissue (Yang et al. 2014), a fall in lactate levels indicative of exhaustion of glycogen stores. In the presence of control aCSF lactate levels were steady at the four temperatures employed (data not shown). Introduction of aglycemia led to a delayed fall in lactate, with minimal effects at 32°C to 37°C, but a delayed fall in lactate occurring at 27°C (Fig. 3B and C). The temperature dependence of the latency to aglycemia‐induced fall in extracellular lactate was consistent with CAP failure (Fig. 3C).

The delay in lactate failure, most apparent at 27°C and illustrated in Figure 3B and C, suggested a decrease in respiration and metabolic rate, which was supported by a temperature dependent fall in oxygen uptake (Fig. 3D).

### Effect of temperature on the CAP profile

**Figure 4:**
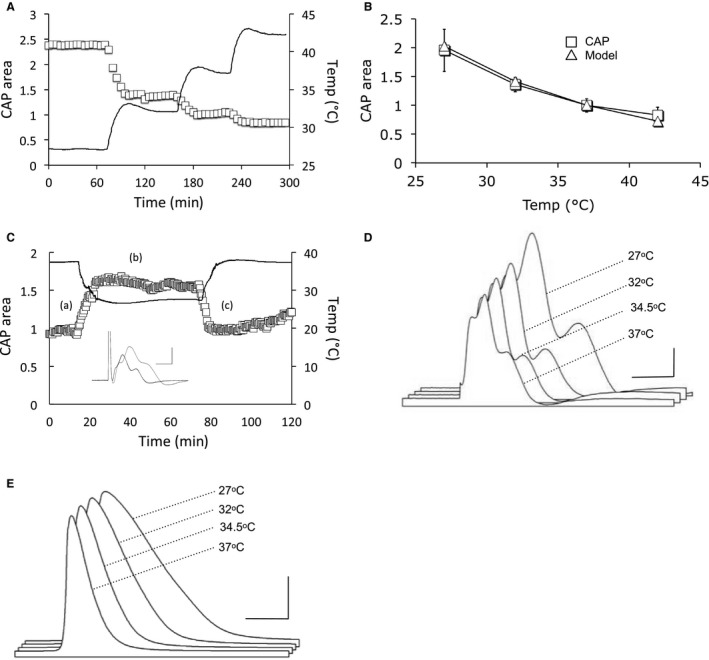
Effect of temperature on CAP profile. (A) CAP area was temperature sensitive, hypothermia resulting in increased CAP area. The open squares denote individual CAP areas (left *y*‐axis), the contiguous line denotes temperature (right *y*‐axis). CAP area was normalized to 1 at 37°C. (B) CAP area (□, *n* = 4) and area of modeled action potentials shown in D (▵) plotted against temperature, normalized to 1 at 37°C, showing an inverse relationship between temperature and CAP area, which is quantified to illustrate the negative correlation (*n* = 4, *P* < 0.0001 two‐tailed Spearman Rank correlation). (C) Switching temperature from 37°C to 27°C for 1 h reversible increased CAP area but had no irreversible effect on the CAP profile. Insert shows CAPs recorded at the time points indicated with overlap between the CAP traces recorded at 37°C. Single trace representative of 4 similar recordings. (D) Example CAPs recorded at indicated temperatures. Scale bars 1 msec and 1 mV. (E) Example simulations of intracellular action potentials modeled at indicated temperatures. Scale bars 1 ms and 40 mV.

In light of our use of the CAP profile as an index of axon conduction, and the exposure of the MONs to varying temperatures, it was necessary to quantify the effect of temperature on the CAP profile by recording the CAP at 27°C then increasing the temperature in 5°C steps to 42°C. Figure 4A clearly shows an inverse relationship between temperature and CAP area. It was also necessary to determine whether switching to hypothermic temperatures for an hour had any long lasting effects on the CAP profile, which would confuse recovery data. Exposing the MON to 27°C for 1 h temporarily increased the CAP area but had no effect on the CAP profile (Fig. 4C). Representative CAPs recorded at four test temperatures are illustrated in Figure 4D, demonstrating that CAP area, amplitude, and duration increase at lower temperatures. It is technically difficult to record intracellular action potentials in MON axons (Gordon et al. 1988) given their small size (mean diameter 0.7 μm) (Allen et al. 2006), thus we carried out computer simulations of intracellularly recorded action potentials from small CNS white matter axons (Kolarik et al. 2013), which show a similar trend to those that contribute to the CAP, namely at higher temperatures the action potential is of smaller amplitude, shorter duration, and smaller area (Fig. 4B and E).

### Hypothermia induced restoration of the CAP

**Figure 5:**
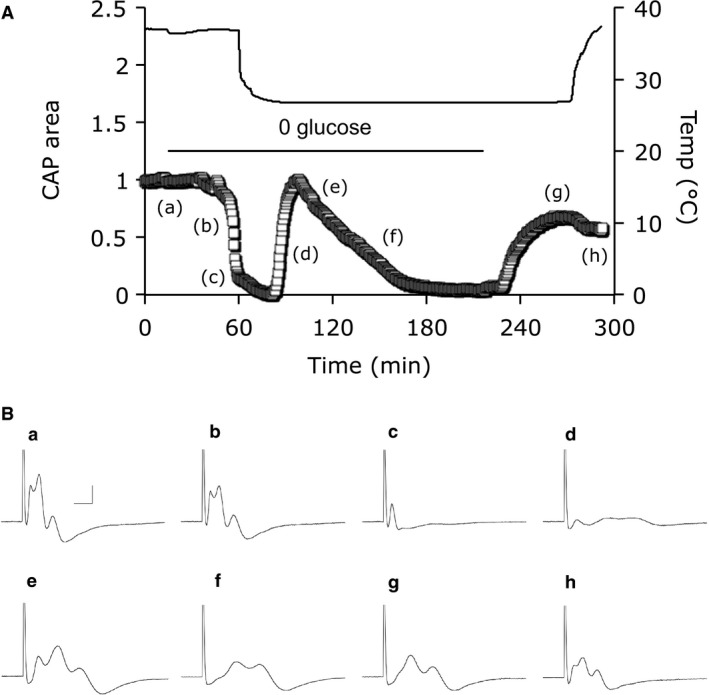
Failing CAP can be restored by rapid exposure hypothermia. (A) On exposure to aglycemia at 37°C there is a delayed failure of the CAP as seen in Figures 1A and 2A. However, upon rapid change of temperature from 37°C to 27°C the CAP, although almost completely lost, suddenly, and rapidly increases temporarily toward baseline before slowly falling during continuous aglycemia at 27°C. Upon reperfusion of control aCSF the CAP partially recovers. (B) Individual CAP profiles at the times indicated in A.

In a MON maintained at 37°C introduction of aglycemia resulted in delayed failure of the CAP as previously described. When the CAP area had fallen to about 10% of baseline and the temperature was rapidly switched to 27°C, there was no immediate effect, with the CAP continuing to fall slowly toward zero. However, about 20 min after switching temperature the CAP area suddenly, and rapidly, increased toward baseline before beginning a slow, inexorable decline toward zero. Upon reperfusion of control aCSF the CAP partially recovered (Fig. 5A and B). These data indicate that it is difficult to exactly determine the point at which glycogen is depleted that is, the onset of phase 3, suggesting it would be difficult to change the temperature during phase 3. Underestimating the duration would lead to the situation illustrated in Figure 5 and overestimating would potentially lead to significant injury occurring after CAP failure but prior to introduction of hypothermia. Thus equivalent experiments of the type designed to investigate the effects of temperature during exposure to anoxia on RON (Fig. 5, Stys et al. 1992a) could not be accurately undertaken and we focused on altering temperature during the reperfusion phase.

### Delayed hypothermia offers neuroprotection after aglycemia

**Figure 6:**
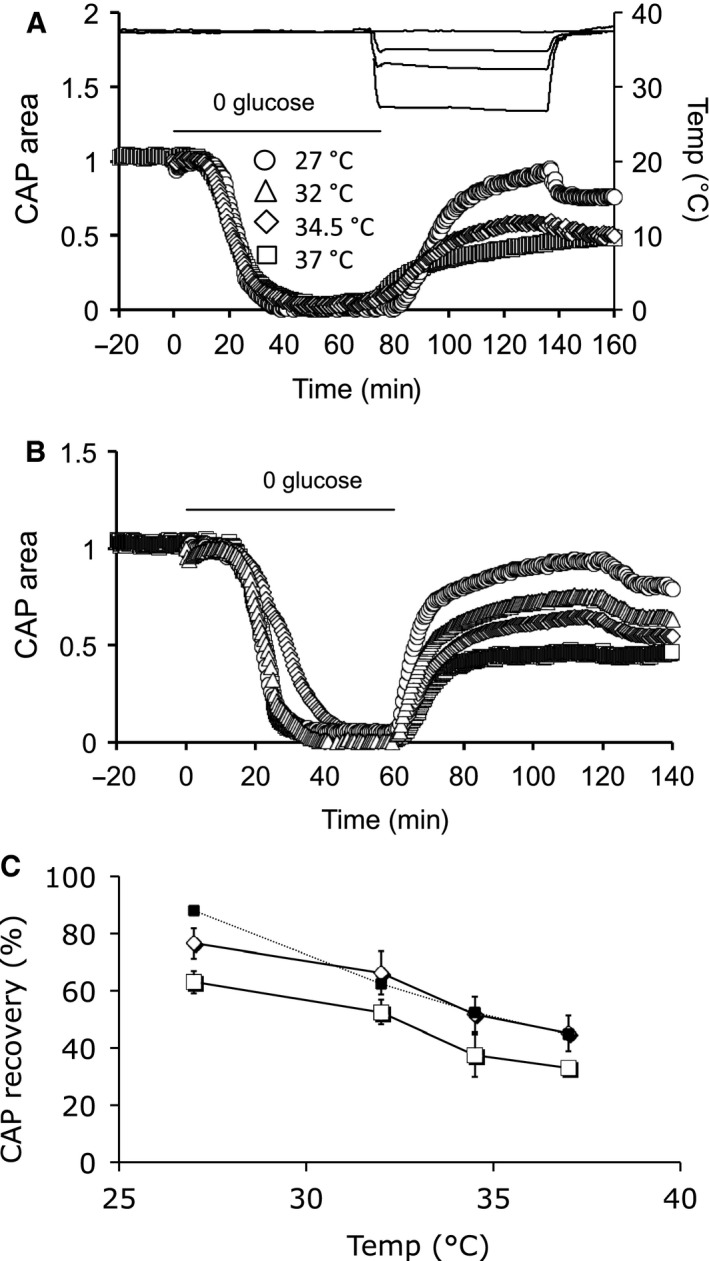
CAP recovery is temperature dependent during reperfusion. (A) MONs were exposed to 75 min of aglycemia (horizontal line from 0 to 75 mins) at 37°C followed by 1 hr of reperfusion at 27°C (○), 32°C (▵), 34.5°C (♢) or 37°C (□). The right axis denotes the temperature recordings (upper black lines). The lower section of the upper horizontal bar denotes the period of temperature change during reperfusion of control aCSF. At 135 min the temperature was switched to 37°C. Following 75 mins of aglycemia hypothermia increased CAP recovery from 33.0 ± 1.2% (*n* = 5) at 37°C to 37.5 ± 7.5% (*n* = 6), 52.5 ± 4.4% (*n*  = 5), or 63.0 ± 4.1% (*n* = 6) at 32°C, 34.5°C or 27°C, respectively. (B) Similar to A except that the exposure to aglycemia at 37°C was for 60 min (horizontal line for 0 to 60 min). Qualitatively similar results were found after 60 min of aglycemia with CAP recovery of 45.0 ± 6.2% (*n* = 7), 51.8 ± 6.3% (*n* = 6), 66.2 ± 7.6% (*n*  = 5), or 76.4 ± 5.2% (*n* = 6) at 37°C, 34.5°C, 32°C or 27°C, respectively. (C) Plot of CAP recovery after 60 min (♢) or 75 min (□) aglycemia followed by reperfusion of control aCSF at temperatures from 27°C to 37°C. The filled squares connected by the dotted line are the data from Figure 2A illustrating CAP recovery when the temperature was altered during the entire experiment. A 2‐way ANOVA detected no significant difference between any pairing of the data ÿ and ♢: *P* = 0.75, but the overall effect of temperature was significant, *P* = 0.001. However, there was a significant difference when comparing □ and ♢: *P* = 0.03, with the overall effect of temperature significant, *P* < 0.0001.

We tested whether hypothermia following aglycemia could afford neuroprotection by imposing either a 60 min or 75 min period of aglycemia at 37°C followed by reperfusion of control aCSF. After reperfusion of control aCSF at the test temperature, the temperature was switched back to 37°C to allow comparison of the CAP areas with the baseline value, which was measured at 37°C. The CAP recovery was temperature dependent (Fig. 6A and C). Qualitatively similar results were found after 60 min of aglycemia (Fig. 6B and C).

### Alkaline aCSF attenuates protection afforded by hypothermia

**Figure 7:**
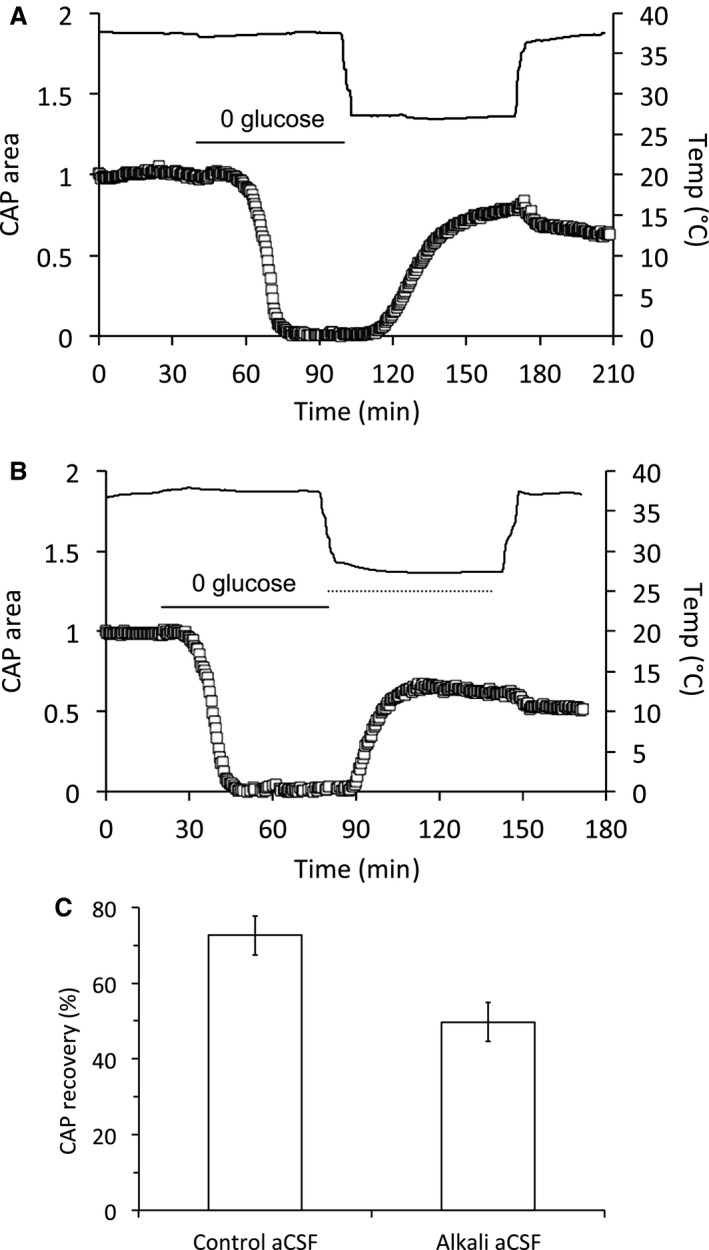
Alkalization of aCSF attenuated hypothermia induced CAP recovery. (A) MONs were exposed to aglycemia for an hour followed by reperfusion for an hour in 10 mM glucose at 27°C. (B) Similar to A with the exception that the aCSF during reperfusion was at pH 7.55 (indicated by the dotted line). (C) Increasing the aCSF pH to 7.55 to offset the hypothermic 0.1 pH unit acidification resulted in an attenuation of CAP recovery, from 72.6 ± 5.2% to 49.7 ± 7.8% (*P* < 0.05, *n*  = 6).

We have previously demonstrated that acidifying the aCSF improved CAP recovery following 90 min of aglycemia as expected from an NMDA receptor‐mediated injury (Yang et al. 2014). We sought to determine any pH related effects imposed by hypothermia on the aCSF. Decreasing the temperature from 37°C to 27°C would be expected to acidify aCSF from pH 7.4 to 7.29 (Chang 1981; Haynes 2010) offering a potential explanation of the neuro‐protective effects of hypothermia. Alkalizing the pH of the aCSF (see Methods) in order to nullify the 0.1 pH unit acidification produced by hypothermia resulted in an attenuation of CAP recovery (Fig. 7A–C).

## Discussion

Mammals evolved to function optimally at a temperature maintained by the organism independent of ambient conditions; in the case of mammals this temperature is 37°C. Such is the sensitivity of proteins to temperature that even slight deviations from 37°C disrupts function, an effect exhibited in the temperature dependent variations in conduction of MON axons, a central white matter tract, where hypothermia decreased conduction velocity but increased compound action potential area. The Q_10_ of most biological systems is between 2 and 3, thus decreases in temperature of only a few degrees can significantly attenuate the metabolic rate, decreasing oxygen consumption and preserving energy substrates. This has been used advantageously to reduce brain injury during the inevitable disruption in delivery of energy substrates to the brain during planned cardiac surgery. Imposing hypothermia on patients suffering from stroke can also reduce injury, thus a therapy that can be imposed *after* the onset of a metabolic insult (stroke, hypoglycemia) would be clinically beneficial given the unpredictable nature of neurological trauma.

### CAP profile and temperature

The CAP profile recorded from the rat optic nerve is temperature dependent with hypothermia promoting elongated CAPs and increased CAP area (Stys et al. 1992a). We demonstrated similar effects in the MON. These results are likely due to the temperature dependence of ion channel kinetics, where hypothermia is associated with slower opening and closing of channels (Hille 2001). We are unable to infer with any confidence whether the greater effect of hypothermia on the repolarizing phase of the action potential is indicative of a heightened sensitivity of K^+^ channels to temperature, but selective ion channel sensitivity to temperature has been reported (Sarria et al. 2012). However, the axoplasmic conductance will also increase with temperature resulting in increased conduction velocity (Moore et al. 1978). The rodent optic nerve membrane potential has recently been shown as temperature sensitive (Coates et al. 2015), but the small temperature range employed in this study would have minimal effects on conduction velocity.

### Aglycemic injury to MON axons

We studied white matter aglycemic injury and found that hypothermia was neuroprotective, suggesting a novel therapy to treat patients suffering from iatrogenic hypoglycemia associated with insulin therapy. Although hypothermia is used to treat patients, the rodent model offers the advantage that we can control the injury process and the onset of the hypothermia. The mammalian nervous system has a high demand for energy substrate in the form of blood borne glucose and oxygen (Clarke and Sokoloff 1999). Numerous studies in the rodent optic nerve have demonstrated the pathology associated with depriving the tissue of oxygen (Stys et al. 1992b), glucose (Brown et al. 2001) or a combination of the two (Fern and Ransom 1997). Initial studies in rat demonstrated a rapid fall in the CAP area on exposure to anoxia followed by irreversible injury (Stys et al. 1992b). Removal of glucose led to a delayed failure of the CAP in MON, endogenous glycogen present in astrocytes acting as an energy buffer to sustain axon conduction in the short term upon removal of glucose, but once the glycogen was depleted conduction failed (Brown et al. 2001). The degree of injury incurred by the MON exposed to aglycemia was dependent upon the duration of exposure to aglycemia (Yang et al. 2014) (Fig. 1). Although exposure to aglycemia for 20 min caused the CAP to fail reperfusion with 10 mM glucose fully restored the CAP indicating that the injury process is initiated after complete CAP failure (phase 1 in Fig. 2C). The failure of the CAP coincides with activation of NMDA receptors on oligodendrocytes by aspartate released from astrocytes (Yang et al. 2014), activation of voltage gated Ca^2+^ channels (VGCC) and reversal of the Na^+^‐Ca^2+^ exchanger (Brown et al. 2001). Each of these pathways leads to toxic Ca^2+^ accumulation in axons (VGCC and Na^+^‐Ca^2+^ exchanger) and oligodendrocytes (NMDA receptors). It should also be noted that the presence of NMDA receptors on oligodendrocytes has been proposed as a mechanism for oligodendrocyte glucose uptake and subsequent shuttling of lactate to axons (Saab et al. 2016). Thus the injury process is activated once the ATP levels fall and disequilibrium of trans‐membrane ion concentrations occurs. In gray matter a large proportion of the injury is incurred during the reperfusion of glucose and is caused by NADPH activation (Suh et al. 2007).

### Mechanism(s) of hypothermic neuroprotection

The role of glycogen in the neuroprotective effects of hypothermia adds a level of complexity that is lacking from the anoxia studies. At the onset of aglycemia glycogen is metabolized to lactate, which fuels axon conduction until glycogen is exhausted. Lowering the temperature would lead to a decreased metabolic rate thus extending the duration in which glycogen supports function. However, this was only seen at the extreme of our range, 27°C. At temperatures of 32°C or 34.5°C there is no significant increase in latency to CAP failure, reflected in the static latency of lactate fall under identical conditions. These data suggest that modest hypothermia has no significant effect on glycogen metabolism, which is corroborated by the small decrease in O_2_ uptake at 32°C. Thus with modest hypothermia any neuro‐protection afforded is not due to a decreased rate of glycogen metabolism.

We have previously shown that at 37°C the CAP recovers to about 49% following 1 h of aglycemia and 1 h of reperfusion in 10 mmol/L glucose. Inhibitors of voltage gated Ca^2+^ channels, Na^+^ Ca^2+^ exchanger or NMDA receptors, improved CAP recovery by about 25% (Brown et al. 2001), 34% (Brown et al. 2001) or 15% (Yang et al. 2014), respectively, indicating there are multiple mechanisms involved in the injury process, although ultimately they all involve intracellular influx of Ca^2+^ (CAP recovers to 100% if Ca^2+^ is excluded from the aCSF) (Brown et al. 2001). Multiple mechanisms have been implied in hypothermic neuroprotection in ischemia, but hypoglycemia is different from both ischemia and anoxia as it is accompanied by an alkalization, rather than an acidification. This alkalization removed the proton block of NMDA receptors and allows their activation by aspartate released from astrocytes. However, hypothermia decreases the pH of aCSF such that our calculations, verified by experimental measurement, show a 0.1 pH unit drop from 7.4 to 7.3 when the temperature is decreased from 37°C to 27°C. This may have profound effects on the involvement of NMDA receptors in the recovery phase following aglycemia, since aglycemic injury is in part due to aspartate activated NMDA receptors. Measurements of aspartate during aglycemia show a delayed elevation above baseline that persists for up to 20 min beyond reperfusion of glucose containing aCSF (Yang et al. 2014). However, the aglycemia‐induced alkalization attenuates rapidly post‐aglycemia and the restoration of a relative acidic interstitial environment would block aspartate‐mediated activation of NMDA receptors. Introduction of aCSF with pH of 7.55 would maintain an alkali environment during reperfusion that would allow continued aspartate‐induced activation of NMDA receptors and may explain why there was little difference between CAP recovery during global hypothermia and exposure to hypothermia during reperfusion. Alternatively the sudden acidification of the interstitial fluid resulting from hypothermia may cause an increased gradient for lactate uptake into axons via monocarboxylate transporter uptake, which co‐transports lactate with H^+^ (Halestrap 2012). The rapid restoration of the CAP after about 80 min in Figure 5A, supports a sudden influx of lactate from the interstitial fluid that temporarily restores CAP condition. However, once the lactate is exhausted it is not replenished and the CAP fails.

### Clinical implications

In comparing the effects of hypothermic neuroprotection on GM and WM it is instructive to appreciate the differences in mechanism, with glutamate and aspartate mediated activation of NMDA receptors occurring in GM, but only aspartate contributing to WM. However, there are additional nonglutamatergic mechanisms in place, namely activation of VGCC and Na^+^‐Ca^2+^exchanger. The alkalization that accompanies aglycemia relieved proton block at the NMDA receptors in WM. However, this effect can be attenuated by alkalizing the aCSF bathing the tissue, which would ensure that the aspartate present during the initial phases of reperfusion would persist in its activation of NMDA receptors, an effect that would be blocked by the rapid restoration of physiological pH on cessation of aglycemia.

## Conflict of Interest

None declared.
